# Human herpesvirus-8 in northwestern China: epidemiology and characterization among blood donors

**DOI:** 10.1186/1743-422X-7-62

**Published:** 2010-03-17

**Authors:** Xing Wang, Bin He, Zhaoxia Zhang, Tao Liu, Hui Wang, Xu Li, Qiong Zhang, Ke Lan, Xiaomei Lu, Hao Wen

**Affiliations:** 1 First Teaching Hospital of Xinjiang Medical University, Urumqi, Xinjiang, PR China; 2 Department of Microbiology and Immunology, College of Medicine, the University of Illinois at Chicago, IL 60612, USA; 3 Blood Center of Urumqi, Xinjiang, PR China; 4 Institut Pasteur of Shanghai, Chinese Academy of Sciences, Shanghai, PR China

## Abstract

**Background:**

Human herpes virus 8 (HHV-8) is the etiologic agent associated with development of classical, AIDS-related, iatrogenic, and endemic Kaposi's sarcoma (KS). Several studies provide strong evidence that HHV-8 can be transmitted by blood transfusion. We evaluated the seroprevalence and potential risk factors of HHV-8 infection in blood donors in one region. We surveyed HHV-8 infection among 4461 blood donors in Xinjiang, China, a unique endemic area for HHV-8 and KS.

**Results:**

The HHV-8 seroprevalence was higher in local minority groups which comprise most KS cases in China, than in Han people. HHV-8 prevalence was 18.6% in the Han ethnic group, 25.9% in Uygur subjects, 29.2% in Kazak subjects, 36.8% in Mongolian subjects, and 21.9% in other ethnic groups. In several subgroups, the time of donation of whole blood seemed to be a risk factor. In HHV-8-seropositive subjects, a larger fraction of local minorities (23.9%) had high HHV-8 titers than that of Han subjects (9.2%). HHV-8 infection was associated with ethnicity and residence.

**Conclusion:**

HHV-8 seroprevalence was significantly high among blood donors in Xinjiang, where the prevalence of KS correlates with HHV-8 prevalence and titers in Uygur and Kazak ethnic groups. Blood exposure represented by the frequency of blood donation indicated a possible blood-borne transmission route of HHV-8 in Xinjiang. Detecting anti-HHV-8 antibodies before donation in this region is therefore important.

## Background

Human herpes virus 8 (HHV-8) is the etiologic agent associated with the development of classical, AIDS-related, iatrogenic, and endemic Kaposi's sarcoma (KS) [1,2]. HHV-8 is also associated with lymphoproliferative diseases, including primary effusion lymphomas and multicentric Castleman's disease [3,4]. Emerging evidence suggests that HHV-8 may be transmitted through sexual contact [5,6], saliva [7], and blood transfusion [8-10]. In the USA, where HHV-8 seroprevalence is low (<10%), HHV-8 is spread by the sexual route, at least among homosexual men [5,6]. In regions or countries with high HHV-8 seroprevalence (>25%), HHV-8 infection increases throughout childhood, suggesting that transmission occurs through saliva or other horizontal routes [11-13]. Of note, HHV-8 infection has been observed in patients who received non-leukocyte-reduced blood [8]. Infectious viruses or viral DNA have been identified from blood donors in the USA and Africa [14,15]. HHV-8 infection has been observed in patients receiving blood transfusions in Uganda, thereby indicating blood-borne transmission of HHV-8 [9,10].

HHV-8 seroprevalence among blood donors varies between different regions. HHV-8 prevalence ranges from 0.2% in Japan, 0-15% in the USA and the UK, up to >50% in some African countries [16,17]. There is a wide range of variations in HHV-8 infection in South America [18]. A few studies focusing on small study populations have been carried out in China. In the inland areas of China, HHV-8 seroprevalence in general population was <8% [19,20]. In Xinjiang, in the northwest of China, HHV-8 seroprevalence ranged from 12.5% to 48% depending on different populations [21-24]. The mode of HHV-8 transmission remains undefined, but the unique pattern of HHV-8 infection in this geographic region correlated well with an increased incidence of KS [21,22,24].

## Results

### Demographic patterns of HHV-8 seroprevalence among blood donors

A total of 4461 serum samples from blood donors were analyzed. Demographic patterns and blood donation-associated behavioral characteristics of HHV-8 infection are shown in Tables 1 and 2, respectively. Overall, 3551 subjects were HHV-8-negative (79.6%) whereas 910 participants were HHV-8-positive (20.4%). In this population, there was no significant difference in HHV-8 seroprevalence with respect to sex, age, marriage, occupation, education, blood type, and times of donation of blood components. Xinjiang residents exhibited HHV-8 seroprevalence of 21.3%, whereas the value for non-residents was 17.7%. The latter were all of Han extraction who had migrated to Xinjiang from inland areas. There was a difference among ethnic groups. HHV-8 seroprevalence in the Han population was lower (18.6%) than in any other ethnic group, such as Uygur (25.9%), Kazak (29.2%), Mongolian (36.8%) and others (21.9%). HHV-8 seroprevalence tended to increase among local minority groups. Most individuals were blood donors, who were negative for hepatitis-B virus (HBV), hepatitis-C virus (HCV), human immunodeficiency virus (HIV), and syphilis (99.8%). Among seven positive subjects for these pathogens, three were HHV-8-positive individuals (42.9%). The relevance of HBV, HCV, HIV, and syphilis to HHV-8 seroprevalence was not further analyzed because the small sample size.

**Table 1:** Sociodemographic characteristics by HHV-8 seroprevalence

Characteristics	Number of subjects (%)	HHV-8 sero-positivity (%)	OR (95% CI)	*p*
Sex			1.1 (0.9-1.2)	0.448
Male	2662 (59.7)	533 (20.0)		
Female	1799 (40.3)	377 (21.0)		
Ethnic background				0.000*
Han	3386 (75.9)	629 (18.6)		
Uygur	526 (11.8)	136 (25.9)	1.5 (1.2-1.9)	0.000*
Kazak	161 (3.6)	47 (29.2)	1.8 (1.3-2.6)	0.001*
Mongolian	87 (2.0)	32 (36.8)	1.0 (1.6-4.0)	0.000*
Other	301 (6.7)	66 (21.9)	2.6 (0.9-1.6)	0.155
Age group (years)				0.777
19-24	2076 (46.5)	430 (20.7)		
24-29	904 (20.3)	166 (18.4)	0.9 (0.7-1.1)	0.141
29-34	590 (13.2)	120 (20.3)	1.0 (0.8-1.2)	0.843
34-39	489 (11.0)	102 (20.9)	1.0 (0.8-1.3)	0.843
39-44	245 (5.5)	56 (22.9)	1.1 (0.8-1.6)	0.436
44-49	105 (2.4)	24 (22.9)	1.1 (0.7-1.8)	0.598
49-54	43 (1.1)	10 (23.3)	1.2 (0.6-2.4)	0.684
>54	9 (0.2)	2 (22.2)	1.1 (0.2-5.3)	0.911
Martial status			1.1 (0.9-1.3)	0.301
Unmarried	3078 (69.0)	615 (20.0)		
Ever married	1383 (31.0)	295 (21.3)		
Occupation				0.208
Soldier	157 (3.5)	25 (15.9)		
Student	1290 (28.9)	276 (21.4)	1.4 (0.9-2.2)	0.112
Professional specialty	740 (16.6)	159 (21.5)	1.4 (0.9-2.3)	0.118
Business\service	697 (15.6)	125 (17.9)	1.0 (0.7-1.8)	0.550
Unidentified job	1577 (35.4)	325 (20.6)	1.3 (0.9-2.1)	0.165
Educational level				0.245
College	1034 (23.2)	217 (21.0)		
Junior College	1080 (24.2)	237 (21.9)	1.0 (0.9-1.3)	0.592
Technical Secondary School	459 (10.3)	79 (17.2)	0.8 (0.6-1.0)	0.092
Senior High School	935 (21.0)	197 (21.1)	1.0 (0.9-1.2)	0.964
Junior High School	844 (18.9)	162 (19.2)	0.9 (0.7-1.1)	0.336
Elementary School	109 (2.4)	18 (16.5)	0.3 (0.3-0.9)	0.273
Residence			1.3 (1.1-1.5)	0.009*
Xinjiang	3321 (74.4)	708 (21.3)		
Outside of Xinjiang	1140 (25.6)	202 (17.7)		
Total	4461 (100.0)	910 (20.4)		

**Table 2:** HHV-8 seroprevalence by blood donor-associated behaviors

Characteristics	Number of subjects (%)	HHV-8 sero-positivity (%)	OR (95% CI)	*p*
Type of blood donation			1.0(0.7-1.4)	0.962
Whole blood	4232 (94.9)	863 (20.4)		
Blood component	229 (5.1)	47 (20.5)		
Time of donation of whole blood				0.845
1	2702(63.8)	557 (20.6)		
2	851 (20.1)	176 (20.7)	1.0 (0.7-1.4)	0.958
3	352 (8.3)	76 (21.6)	0.0 (0.7-1.4)	0.758
4	168 (4.0)	25 (14.9)	1.1 (0.7-1.6)	0.151
5	67 (1.6)	13 (19.4)	0.7 (0.4-1.2)	0.841
6	39 (0.9)	7 (17.9)	0.9 (0.5-1.9)	0.711
7	25 (0.6)	4 (16.0)	0.8 (0.4-2.0)	0.593
8	28 (0.7)	5 (17.9)	0.7 (0.2-2.3)	0.740
Time of donation of blood components				0.678
1-5	64 (27.9)	13 (20.3)		
6-10	29 (12.7)	10 (34.5)	1.0 (0.5-1.8)	0.067
12-15	33 (14.4)	6 (18.2)	2.1 (1.0-4.4)	0.987
16-20	24 (10.5)	4 (16.7)	0.9 (0.4-2.1)	0.754
21-25	19 (8.3)	2 (10.5)	0.8 (0.3-2.3)	0.652
26-30	26 (11.4)	5 (19.2)	0.5 (0.1-2.0)	0.299
31-40	20 (8.7)	3 (15.0)	0.9 (0.7-2.5)	0.884
41-44	14 (6.1)	4 (28.6)	0.7 (0.2-2.4)	0.553
Pathogens screen			0.0 (0.0-1.0)	0.982
Seronegative	4452 (99.8)	907 (20.4)	2.9(0.7-13.1)	0.159
Seropositive	7 (0.2)	3 (42.9)		
(HBV/HCV/HIV/syphilis)				

### Assessment of risk factors

The univariate associations between HHV-8 seropravelence and subject characteristics are illustrated in Tables 1 and 2. Ethnic background was found to be associated with HHV-8-positive status. This variable exhibited a statistically significant difference whereby the odds ratio (OR) was high for Uygur (1.5, 95% confidence interval (CI) 1.2-1.9, p < 0.000) and Kazak (1.8, 95% CI 1.3-2.6, p < 0.001) ethnic groups. Residence appeared to be associated with HHV-8 infection (OR = 1.3, 95% CI 1.1-1.5, p < 0.009). No associations were observed between HHV-8 seroprevalence and sex, age, education, marital status, occupation and blood donation-associated behaviors. To further identify independent risk factors, all variables from the univariate analysis were entered into multiple logistic regression models (Table 3). In this analysis, HHV-8-positive status was associated with Uygur (OR = 1.4, 95% CI 1.1-1.9, p < 0.000) and Kazak (OR = 1.8, 95% CI 1.2-2.6, p < 0.000) ethnic groups. A strong association of HHV-8 infection was seen with the Mongolian (OR = 2.7, 95% CI 1.7-4.2, p < 0.000) ethnic group. With the increasing frequency of donation of whole blood, the possibility of infection also increased in several subgroups such as 3 (OR = 1.9, 95% CI 1.1-3.2, p < 0.021), 5 (OR = 3.5, 95% CI 1.3-9.7, p < 0.016), 6 (OR = 4.6, 95% CI 1.2-17.8, p < 0.025), and 8 (OR = 5.1, 95% CI 1.1-23.9, p < 0.04). There was no association between HHV-8 seroprevalence and the other variables evaluated in Tables 1 and 2 (data not shown).

**Table 3:** Analyses by risk factor

	OR (95% CI)	p
Ethnic background		0.000*
Han	1.0	
Uygur	1.4 (1.1-1.9)	0.004*
Kazak	1.8 (1.2-2.6)	0.004*
Mongolian	2.7 (1.7-4.2)	0.000*
Other	1.3 (0.9-1.7)	0.142
Time of donation of whole blood		0.185
1	1.0	
2	1.2 (0.9-1.7)	0.291
3	1.9 (1.1-3.2)	0.021*
4	1.5 (0.7-3.0)	0.278
5	3.5 (1.3-9.7)	0.016*
6	4.6 (1.2-17.8)	0.025*
7	4.1 (0.8-20.7)	0.089
8	5.1 (1.1-23.9)	0.040*

### HHV-8 antibody titers in HHV-8-positive individuals

**Figure 1:**
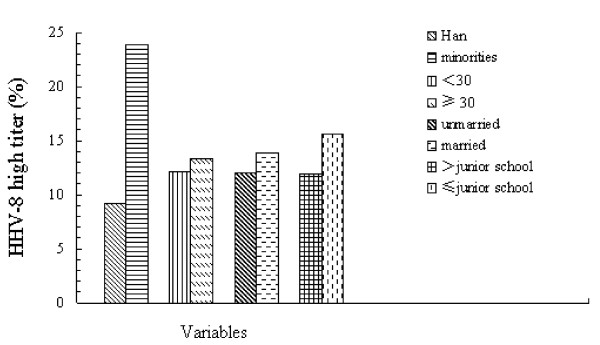
**Relative HHV-8 high titers in different subgroups among blood donors**.

We compared relative levels of HHV-8 antibody titers among HHV-8-positive subjects. High titers were noted in: 12.2% of those aged <30 years, 13.4% of subjects aged >30 years, 12% of unmarried individuals, and 13.9% of married individuals (Figure 1). There was no major difference in these subgroups. Among study subjects, 11.9% of individuals who had a Junior High School-education had high titers. This number increased to 15.6% for those who studied beyond Junior High School. Only 9.2% of HHV-8-positive subjects from the Han ethnic group had high titers. This number increased to 23.9% in minority groups which included the Uygur and Kazak ethnic groups. As compared with subjects from the Han ethnic group, local minorities had a larger proportion of individuals with a higher level of HHV-8 antibody titers.

**Figure 2:**
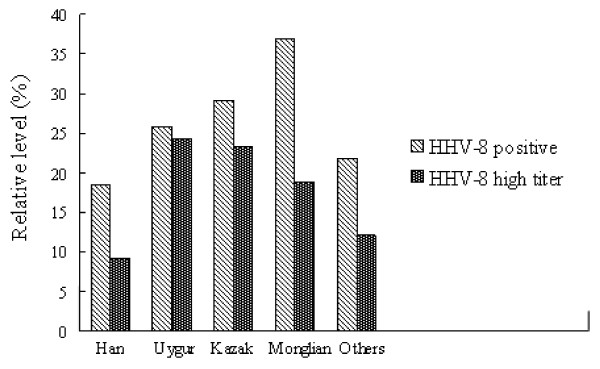
HHV-8 infection by seroprevalence and high antibody titers in different ethnic groups

We further examined HHV-8 antibody titers in each individual ethnic group. The Han group exhibited a HHV-8 seroprevalence of 18.6%, but only 9.2% of HHV-8-positive subjects had high anti-HHV-8 titers (Figure 2). A similar trend was seen in the Mongolian group and other groups except the Uygur and Kazak groups. The Mongolian group showed a HHV-8 seroprevalence of 36.8%, whereas 18.8% had high HHV-8 antibody titers. Other groups showed a HHV-8 seroprevalence of 21.9%, whereas 12.1% had high HHV-8 titers. Strikingly, different results were seen in Uygur and Kazak groups; 24.3% of the Uygur group with HHV-8 infection had high HHV-8 titers, and 23.4% of Kazak who were HHV-8 positive had high HHV-8 titers. Therefore, a larger fraction of Uygur and Kazak groups had higher HHV-8 antibody titers than those in the other ethnic groups.

## Discussion

The present study was the first large-scale survey of HHV-8 seroprevalence in blood donors in China. Recent reports showed that HHV-8 seroprevalence was 7.3% in Liaonin province and 5.7% in Shandong province among healthy blood donors [19,25]. These studies focused on the Han ethnic group, and the study population was small. A study in the Han ethnic group in Hubei province revealed that HHV-8 seroprevalence was 5.2% [20]. The prevalence of HHV-8 infection reported in these studies was similar to those among the adult population in North America [6,14,26]. These observations suggest that HHV-8 seroprevalence in China was, in general, low. The Xinjiang area, located in the northwest of China, exhibited a distinct pattern. We noted that HHV-8 seroprevalence was relatively high in the Han ethnic group living in Xinjiang: 18.6% of blood donors were HHV-8 positive. Moreover, HHV-8 seroprevalence was 17.7% among the 1140 non-residential Han ethnic group who migrated to Xinjiang from inland areas. Thus, an elevated HHV-8 seroprevalence in the Han ethnic group in Xinjiang province implies an association of HHV-8 infection with the living environment. These potential factors which contribute to the differences within the same ethnic group suggest an increased opportunity for members of the Han ethnic group to have close contact with highly infected populations. And the relatively low level of public health in Xinjiang province due to the economic situation in this region may benefit the spread or transmission of HHV-8, but this hypothesis needs conformation. The precise impact of being resident in Xinjiang on HHV-8 infection could not be ascertained because of the cross-sectional nature of the present study.

The present study suggested that HHV-8 seroprevalence was associated with ethnicity but not with sex, age, marital status, occupation, educational level, blood type, and time of donation of blood components. Among blood donors, HHV-8 seroprevalence was 18.6% in the Han group, 25.9% in the Uygur group, 29.2% in the Kazak group, and 36.8% in the Mongolian group. When combined, the mean prevalence of HHV-8 infection in local minorities was 30.6%. Compared with Han subjects living in Xinjiang, the elevated HHV-8 seroprevalence in local minorities was significant. Hence, what was the basis for the observed differences? It is known that local minorities have resided in Xinjiang for generations, and that they have unique cultural practices. An intriguing possibility is that different cultural practices or social behaviors may play a part in HHV-8 infection. Alternatively, genetic factors may affect the susceptibility to HHV-8 infection. Additional studies are required to address these issues.

Several studies have demonstrated that HHV-8 seroprevalence is correlated with the occurrence of KS in Europe and Africa [27-30]. In Ghana and Egypt, a high prevalence of HHV-8 does not correlate well with KS onset [11,31]. HHV-8 seroprevalence is high among Amerindians in Brazil, French Guiana and Ecuador [32-34]. Nonetheless, KS has not been reported for these populations. The present study revealed that HHV-8 seroprevalence was higher in Han subjects residing in Xinjiang than in the inland areas of China. Furthermore, it was higher in local minority groups than in Han subjects. HHV-8 prevalence remained high in blood donors residing in Xinjiang, but classical KS and AIDS-related KS were observed only among local minority groups [21,22]. Taken together, these data support the hypothesis that additional factors other than HHV-8 may be involved in KS development.

Several researchers reported blood-borne transmission of HHV-8 among a large cohort of drug users, or prospective studies on blood-transfusion recipients [8,10]. The present study was in accordance with these studies because it established a positive relationship between an increasing prevalence of infection with the corresponding frequency of donation of whole blood. To a certain extent, the time of donation represents the opportunity of blood exposure. In China, laws pertaining to donation of whole blood were passed in 1992. Before 1992, non-standard protocols for blood collection, and absence of instruments and materials were common. These conditions could increase the prevalence of HHV-8 infection by blood exposure. A possible explanation for the contradictory results in the univariate analysis with respect to the time of donation of blood components may be due to the small number of samples in each group or undetermined factors.

There was a difference in HHV-8 antibody titers among HHV-8-seropositive individuals in the present study. Specifically, a larger fraction of local minorities had high HHV-8 antibody titers than Han subjects. This was evident in the Uygur and Kazak ethnic groups. Whether this reflects enhanced replication of HHV-8 or enhanced immune responses is not clear. Notably, KS patients are seen in Uygur and Kazak ethnic groups in Xinjiang hospitals [21,22]. The patients are typically elderly men who have multiple nodular lesions in the lower or upper extremities. Intriguingly, KS patients tend to be younger among those who are also infected with HIV. Hence, high HHV-8 antibody titers in Uygur and Kazak groups appeared to correlate with the development of KS in these ethnic groups.

## Conclusions

HHV-8 seroprevalence was significantly high in blood donors from Xinjiang. A critical question relevant to public health is if HHV-8 is transmitted through blood transfusion in the Xinjiang area. Several studies in the USA and Africa suggest an association between HH-8 infection with blood transfusion **Error! Reference source not found**. Given that HHV-8 seroprevalence in the Xinjiang area is high, assessing the risk of HHV-8 transmission via blood transfusion in future studies is essential.

## Methods

### Ethical approval of the study protocol

The present study was approved by the Institutional Ethics Committee of the First Teaching Hospital of Xinjiang Medical University (Urumqi, Xinjiang, China). Written informed consent was obtained from all subjects, and patient confidentiality was ensured.

### Study population

A cross-sectional study was designed to assess the seroprevalence of HHV-8 infection among blood donors in Xinjiang, China. Serum samples, collected and deposited from all five blood banks belonging to Xinjiang Blood Center between August 2006 and May 2007, were analyzed. These samples were derived from 4832 blood donors belonging to different ethnic groups: Han, Uygur, Kazak, Mongolian, and others. All samples went through a standard screening for HBV, HCV, HIV, and syphilis. A questionnaire regarding age, sex, ethnicity, marital status, education and residence was collected. Of 4832 blood donors, 4461 blood donors completed all sections of the questionnaire. Serum samples were centrifuged and stored at -80°C before HHV-8 serologic testing.

### Laboratory procedures

The coded serum specimens were tested for HHV-8 antigens of ORF_73_, ORF_65 _and K8.1 using enzyme-linked immunosorbent assays as described ^22,24 ^**Error! Reference source not found**. This assay has a sensitivity of 82% and a specificity of 96% ^22 ^. Briefly, viral antigen-coated plates were incubated with serum samples diluted at 1:100. This was followed by incubation with goat anti-human immunoglobulins (including IgG) conjugated with horseradish peroxidase (HRP; Tago Immunologicals). The mean optical density at 450 nm was determined. Controls included serum samples derived from KS patients and HHV-8-negative individuals. Based on the assays from control groups, the HHV-8-positive cutoff was set to the value that was three-times that of the negative control. The HHV-8 high titer was set to the value that was more than five-times that of the negative control.

### Statistical analyses

All statistical analyses were carried out using SPSS software version 12.0 (SPSS Incorporated, Chicago, IL, USA). The univariate analysis of categorical variables was evaluated by #2 test with *P *< 0.05 being considered significant. Associations revealed by OR and P were evaluated at a CI of 95%. Multivariate logistic regression analysis was carried out to control for confounding factors. CI was calculated based on coefficients and standard errors from the logistic model. Seropositive prevalence and risk factors were compared between groups.

## Competing interests

The authors declare that they have no competing interests.

## Authors' contributions

XW carried out study design, sample collection, and statistical analyses performance; she also participated in antibody detection. ZZ, TL, QZ and HW also participated in antibody detection. XLi and XLu wrote and collected the questionnaire. BH drafted the manuscript. KL participated in the design of the study and carried out statistical analyses.

HW conceived the study, and participated in its design and coordination; he also helped to draft the manuscript. All authors read and approved the final manuscript.
